# Design and Embedded Implementation of Secure Image Encryption Scheme Using DWT and 2D-LASM

**DOI:** 10.3390/e24101332

**Published:** 2022-09-22

**Authors:** Heping Wen, Zefeng Chen, Jiehong Zheng, Yiming Huang, Shuwei Li, Linchao Ma, Yiting Lin, Zhen Liu, Rui Li, Linhao Liu, Wenxing Lin, Jieyi Yang, Chongfu Zhang, Huaide Yang

**Affiliations:** 1 School of Electronic Information, Zhongshan Institute, University of Electronic Science and Technology of China, Zhongshan 528402, China; 2 School of Information and Communication Engineering, University of Electronic Science and Technology of China, Chengdu 611731, China; 3 School of Electronic Information, Dongguan Polytechnic, Dongguan 523808, China

**Keywords:** image encryption, DWT, frequency-domain analysis

## Abstract

In order to further improve the information effectiveness of digital image transmission, an image-encryption algorithm based on 2D-Logistic-adjusted-Sine map (2D-LASM) and Discrete Wavelet Transform (DWT) is proposed. First, a dynamic key with plaintext correlation is generated using Message-Digest Algorithm 5 (MD5), and 2D-LASM chaos is generated based on the key to obtain a chaotic pseudo-random sequence. Secondly, we perform DWT on the plaintext image to map the image from the time domain to the frequency domain and decompose the low-frequency (LF) coefficient and high-frequency (HF) coefficient. Then, the chaotic sequence is used to encrypt the LF coefficient with the structure of “confusion-permutation”. We perform the permutation operation on HF coefficient, and we reconstruct the image of the processed LF coefficient and HF coefficient to obtain the frequency-domain ciphertext image. Finally, the ciphertext is dynamically diffused using the chaotic sequence to obtain the final ciphertext. Theoretical analysis and simulation experiments show that the algorithm has a large key space and can effectively resist various attacks. Compared with the spatial-domain algorithms, this algorithm has great advantages in terms of computational complexity, security performance, and encryption efficiency. At the same time, it provides better concealment of the encrypted image while ensuring the encryption efficiency compared to existing frequency-domain methods. The successful implementation on the embedded device in the optical network environment verifies the experimental feasibility of this algorithm in the new network application.

## 1. Introduction

With the rise of big data and the development of digital image processing technology [1,2,3,4,5], the digital image as an important transmission medium, contains a large amount of important data, such as personal privacy and confidential information. The importance of secure transmission is self-evident. However, in contrast to textual information, digital image information is characterised by high complexity and strong pixel correlation [6,7,8]; therefore, it is essential to study encryption algorithms for digital images.

In order to realize the secure transmission of digital images, many new encryption schemes have been proposed. Among them, the problem of encrypted image transmission is particularly important [9,10]. Optical network technology based on fibre optic communication has developed rapidly in recent years, and due to the advantages of efficient transmission in optical networks, it plays an irreplaceable role in the transceiver side of embedded devices [11,12].

However, on the one hand, many such algorithms do not have provable security, and on the other hand, they are less combined with Optical Access Network communication, which is also an important problem that must be solved in applications [13]. Therefore, it is necessary to study image encryption and transmission technologies based on optical networks. This can show that image encryption and transmission technology based on all-optical networks in the context of the big data era have certain theoretical value and practical significance [14,15].

Throughout the international research status, the research popularity of image encryption technology is increasing, and various encryption methods to enhance the security of algorithms have been proposed [16,17,18,19,20]. In 2020, Ref. [21] proposed cryptanalysis of an image block encryption algorithm based on chaotic maps.Its equivalent secret key can be easily recovered with some chosen plain-images. The summarized security defects can be used to inform designers of image-encryption algorithms about common security pitfalls in the field of image security, particularly chaotic cryptography.

In the same year, Ref. [22] proposed a new image multi encryption algorithm based on HDWT hyper-chaotic system generation, which increased the number of sequence generators and increased the size of the key space exponentially. In the same year, Ref. [23] proposed a novel time-lagged chaotic system, and a novel digital image-encryption algorithm was designed based on this system. The experimental results showed that the algorithm has the advantages of good encryption effect and high system security.

In 2021, Ref. [24] proposed an image-encryption algorithm based on DNA encoding and two specially configured binary chaotic kernels. After the security analysis of the scheme, it was proven that the algorithm can resist known attacks and has excellent encryption performance. In 2022, Ref. [25] re-analysed the theoretical security and practical performance of a medical privacy protection scheme based on DNA en-coding and chaotic maps. Detailed experimental results were provided to show more security defects, including the existence of a large number of weak secret keys, weak key sensitivity, and low efficiency.

The DNA-based encryption scheme that was analysed is important for promoting interdisciplinary research on application of DNA computing in cryptography. Most studies on encryption algorithms have achieved good results [26,27,28,29]; however, in the current research, the images are regarded as two-dimensional matrix encryption, which exposes two defects:

(1) Most image encryption is only based on spatial-domain algorithms, and the encryption speed is slower. Compared with the spatial-domain algorithm, the frequency-domain algorithm has higher encryption efficiency. Due to the complexity of the frequency-domain algorithm, it can bring greater deciphering difficulties to illegal decipherers, and the security performance is much higher than that of the spatial-domain algorithm. (2) The system based on chaos is relatively complex. Although it ensures the randomness of chaotic sequences, there are still problems, such as high algorithm redundancy and slow generation of chaotic systems.

Compared with the existing research, this paper proposes a frequency-domain image-encryption algorithm based on two-dimensional chaos and discrete wavelet transform and makes innovative research. First, a dynamic key with plaintext correlation is generated by MD5, and 2D-LASM chaos is generated based on the key to obtain a chaotic pseudo-random sequence. Secondly, the plaintext image is transformed by DWT to map the image from the time domain to the frequency domain and decompose it into low-frequency (LF) coefficient and high-frequency (HF) coefficient.

Then, the chaotic sequence is used to encrypt the LF coefficient with the structure of “confusion-permutation”, and only the HF coefficient is scrambled. The processed LF coefficient and HF coefficient are reconstructed to obtain the frequency-domain ciphertext image. Finally, the ciphertext is dynamically diffused using the chaotic sequence to obtain the final ciphertext. Theoretical analysis and simulation experiments show that the algorithm has a large key space and can effectively resist various attacks.

At the same time, it reduces the burden of channel transmission by reducing the redundancy of image data and ensures the security of image transmission in the public channel. In addition, combined with the characteristics that wavelet transform is suitable for real-time applications [30], we studied an end-to-end image security system based on Raspberry Pi and transplanted the algorithm to the embedded system equipment to run. The experimental results show that this system can protect user data better in real-time transmission under the transport layer TCP protocol and has good application prospects and research value in the field of information security.

## 2. Related Theory

### 2.1. 2D-Logistic-Adjusted-Sine Map

The chaotic system first proposed by the American meteorologist Lorenz is a nonlinear dynamical system with the characteristics of non-divergence, non-convergence and non-period. Due to the complex dynamics of chaotic systems, the sequences generated by these systems are usually strongly random [31,32,33,34]. At the same time, due to the high initial value sensitivity of chaotic systems, the sequences are usually difficult to predict; therefore, chaotic sequences have been widely used in secure communication.

**Figure 1:**
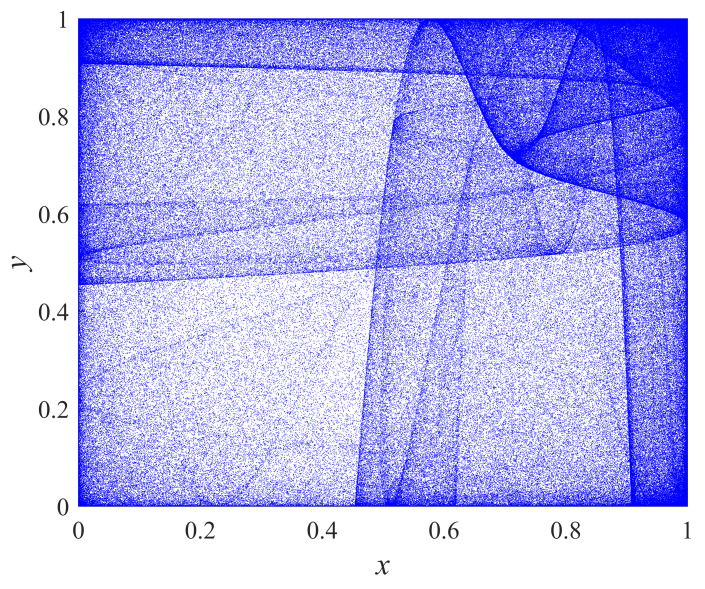
Phase diagrams of 2D-LASM with μ = 0.85.

Ref. [35] indicated that the key size of image-encryption algorithms should be at least $10^{30}\approx 2^{100}$. Compared with two-dimensional chaotic systems, one-dimensional chaotic systems, such as Logistic and Sine are easy to predict the iterative sequence, have a small key space, and do not have complex chaotic properties. The high-dimensional chaotic systems formed by the combination of low dimensional chaotic systems have more control parameters, and the chaotic structures are more complex. In this paper, we use the 2D-LASM designed by Hua et al. [36] based on the combination of Logistic and Sine mapping chaotic system, which is expressed as

(1) $$\left\{\begin{array}{c} x_{n+1}=\sin\left(\pi \beta {}_{1}\left(y_{n}+3\right)x_{n}\left(1-x_{n}\right)\right)\\ y_{n+1}=\sin\left(\pi \beta {}_{2}\left(x_{n}+3\right)y_{n}\left(1-y_{n}\right)\right) \end{array}\right.$$

where $x_{n+1},y_{n+1}\in \left[0,1\right]$ are the pseudo-random sequences generated by chaos and the control parameters $\beta_{1},\beta_{2}\in \left[0,1\right]$. The two input parameters of this chaotic system interact with each other, and output pair $\left(x_{n}+1,y_{n}+1\right)$ is distributed to the two-dimensional phase plane. Figure 1 is the phase diagram of 2D-LASM. As can be seen from the phase diagram, the output sequence $\left(x_{i},y_{i}\right)$ of the 2D-LASM map covers a large area on a two-dimensional plane.

**Figure 2:**
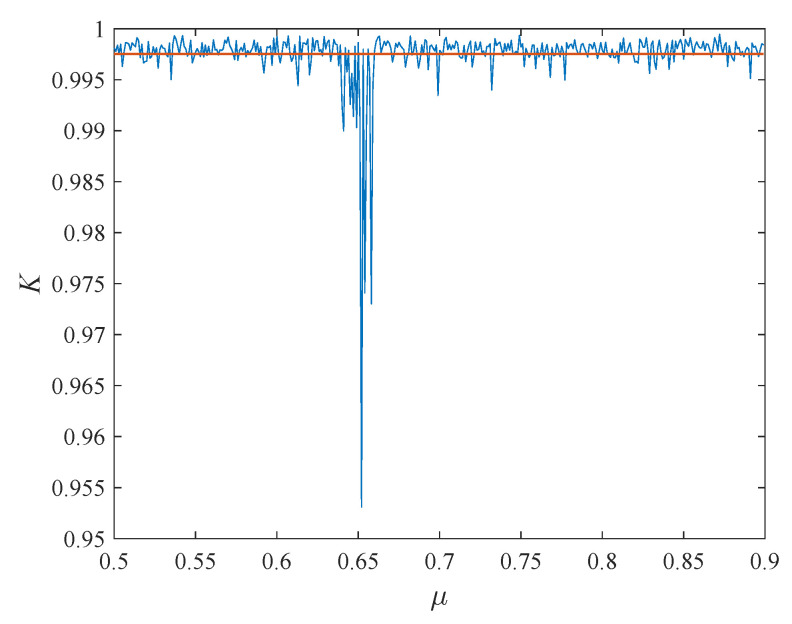
The 0–1 Gottwald–Melbourne test.

The 0–1 Gottwald–Melbourne test can determine the regular motion and chaotic motion by calculating the parameter *k* asymptotically close to 0 or 1. As shown in Figure 2, the *k* value of the average result of 10,000 times is 0.9975, which is close to the theoretical value [37]. This can verify the excellent performance of the chaotic system.

### 2.2. Discrete Wavelet Transform

Wavelet transform performs multi-scale refinement of signal gradually through scaling and translation operations [38,39], finally achieves time subdivision at high frequency and frequency subdivision at low frequency, and can automatically adapt to the requirements of time–frequency signal analysis.

**Definition** **1.** *For any* $f\left(t\right)\in L^{2}\left(R\right)$ *, after the basic wavelet* ψ(t) *is shifted by* b *, and then the inner product is made with the signal to be analysed* f(t) *at different scales* a *. The mathematical expression is given by*

(2) $$WT_{f(a,b)}=\frac{1}{\sqrt{a}}\int_{R}^{}f\left(t\right)\psi \left(\frac{t-b}{a}\right)dt\;a,b\in R,a>0$$

*where a is the scale factor, whose role is to stretch the basic wavelet* ψ(t) *function, b is the translation factor, whose value can be positive or negative, and a and b are both continuous variables; thus, it is called a continuous wavelet transform.*

**Definition** **2.** *The DWT is to discretize the scale factor a and the translation factor b on the basis of the continuous wavelet transform, and turn it into a power series structure,*$a=a_{0}^{j}\left(a_{0}\neq 1,j\in Z\right),b=ka_{0}^{j}b_{0}\left(b_{0}>0,k\in Z\right)$*, the mathematical expression for the 2D-DWT of the image f(x,y) of size*M×N*is*

(3) $$\left\{\begin{array}{c} W_{\phi }\left(j_{0},a,b\right)=\frac{1}{\sqrt{MN}}\sum_{x=0}^{M-1}\sum_{y=0}^{N-1}f\left(x,y\right)\phi {}_{j_{0},a,b}\left(x,y\right)\\ W_{\psi }^{i}\left(j,a,b\right)=\frac{1}{\sqrt{MN}}\sum_{x=0}^{M-1}\sum_{y=0}^{N-1}f\left(x,y\right)\psi^{i}{}_{j,a,b}\left(x,y\right),i=\left\{H,V,D\right\} \end{array}\right.$$

*where*$j_{0}$*is an arbitrary scale initial value, i is the superscript of the assumed values H, V and D,*$\phi_{j_{0},a,b}\left(x,y\right)$*represents the scaling function,*$W_{\phi }\left(j_{0},m,n\right)$*is*f(x,y)*approximate coefficients at the scale*$j_{0},W_{\phi }\left(j_{0},m,n\right)$*coefficients add detail coefficients in the horizontal, vertical, and diagonal directions for scale*$j\geq j_{0}$.

**Figure 3:**
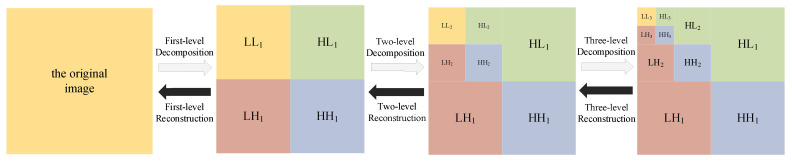
Block diagram of the three-level DWT scheme.

The schematic diagram of the image wavelet decomposition is shown in Figure 3. LL denotes low frequency, HL, LH, and HH denote high frequency, and the subscripts 1 and 2 denote the first-level and second-level decomposition, respectively. The image is decomposed into four sub-images after 2D-DWT: the low-frequency component of the original image, the high-frequency component in the horizontal direction, the high-frequency component in the vertical direction, and the high-frequency component in the diagonal direction. The low-frequency components continue to be decomposed into sub-images of lower resolution in the next level in exactly the same way.

**Figure 4:**
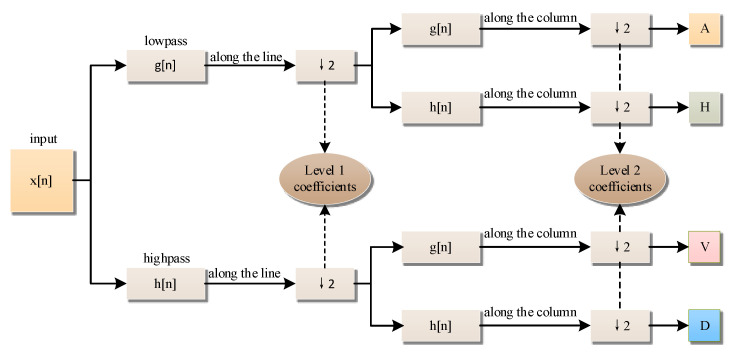
Schematic diagram of wavelet decomposition data flow.

In this way, the image is decomposed into multiple sub-images at different resolution levels and in different directions, which is consistent with the visual characteristics of the human eye. The schematic diagram of the wavelet decomposition data flow is shown in Figure 4. x[n] represents the discrete input signal, g[n] represents the low-pass filter, which is used to retain the low-frequency components of the input signal and remove the high-frequency components, h[n] represents the high-pass filter, whose function is opposite to that.

**Figure 5:**
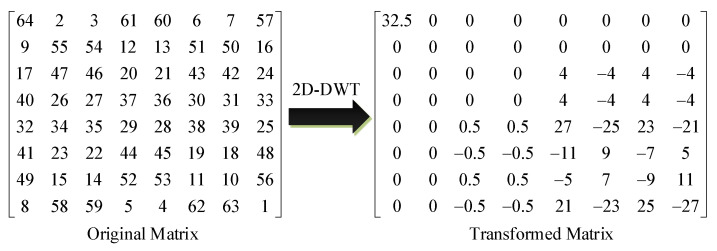
Data comparison before and after 2D-DWT.

The 2D-DWT processing is performed on the 8 × 8 image block by transforming each row of the array, and then transforming each column of the array after the row transformation. Finally the transformed image data array is encoded. The elements in the upper left corner are called low-frequency coefficients, and the remaining elements are called high-frequency coefficients. The data before and after the transformation is shown in Figure 5.

Among them, the data 32.5 of Transformed Matrix is the low-frequency coefficients of the matrix, which shows that the energy of the image is mainly concentrated in the low-frequency coefficients after 2D-DWT. Based on this, only the low-frequency coefficients in the frequency domain after DWT need to be encrypted to obtain a more satisfactory image encryption effect.

## 3. Design of Encryption Algorithm

**Figure 6:**
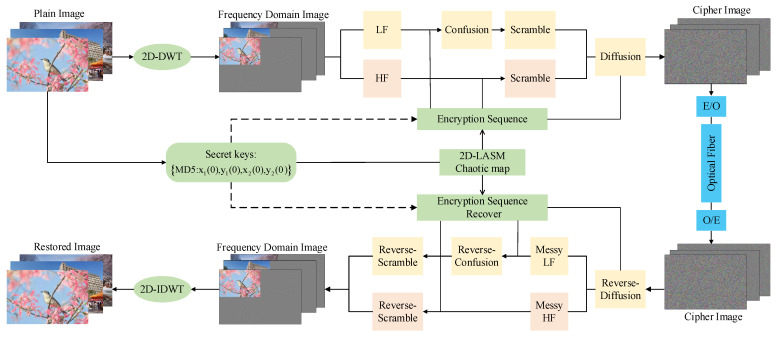
Principles and mechanisms of image encryption and decryption.

In traditional encryption algorithms, they are often designed for one-dimensional data stream information, the drawbacks of which are high computational complexity and low encryption efficiency, which are slightly stretched for digital images with large data volume, spatial order, strong correlation, and high redundancy. In particular, with the development of technology, the information contained is even gradient increasing. Therefore, this paper proposes an image-encryption algorithm based on DWT and a 2D-LASM chaotic system and introduces a dynamic key with plaintext correlation to achieve “one-time pad” encryption, and the specific process of encryption and decryption is shown in Figure 6. The specific encryption algorithm is designed as follows.

**Step 1**: Use 2D-DWT to decompose plaintext images

The image is decomposed of DWT into four subbands: LL,LH,HL, and HH, are performed according to Equation (4), and the coefficients of each subband are calculated using a Haar filter to map the image matrix from the spatial domain to the frequency domain. Then, the data is processed into the pixel value range of 0–255 to obtain one low-frequency image and three high-frequency images. The specific treatment is shown as

(4) $$\left\{\begin{array}{c} LL\left(x,y\right)=\frac{p(x,y)+p(x,y+1)+p(x+1,y)+p(x+1,y+1)}{2}\;\\ LH\left(x,y\right)=\frac{p(x,y)+p(x,y+1)-p(x+1,y)-p(x+1,y+1)}{2}\;\\ HL\left(x,y\right)=\frac{p(x,y)-p(x,y+1)+p(x+1,y)-p(x+1,y+1)}{2}\;\\ HH\left(x,y\right)=\frac{p(x,y)-p(x,y+1)-p(x+1,y)+p(x+1,y+1)}{2} \end{array}\right.$$

where p(x,y), p(x,y+1), p(x+1,y), and p(x+1,y+1) are the four pixel points of the LL,LH,HL, and HH.

**Step 2**: Pseudo-random sequence preprocessing

The key of this algorithm consists of the MD5 value of the plaintext image, the initial value of the 2D-LASM chaos, and the control parameters. MD5 can scramble the initial values of the chaotic system to make the key sequence more sensitive to the plaintext, thus, enhancing the security of the algorithm. The detail of this algorithm is shown as

(5) $$\left\{\begin{array}{c} x_{1}^{{}^{\prime }}\left(0\right)=x_{1}\left(0\right)+\left(m_{1}⊕m_{2}⊕m_{3}⊕m_{4}\right)/256\\ y_{1}^{{}^{\prime }}\left(0\right)=y_{1}\left(0\right)+\left(m_{5}⊕m_{6}⊕m_{7}⊕m_{8}\right)/256\\ x_{2}^{{}^{\prime }}\left(0\right)=x_{2}\left(0\right)+\left(m_{9}⊕m_{10}⊕m_{11}⊕m_{12}\right)/256\\ y_{2}^{{}^{\prime }}\left(0\right)=y_{2}\left(0\right)+\left(m_{13}⊕m_{14}⊕m_{15}⊕m_{16}\right)/256 \end{array}\right.$$

where ⊕ is a bitwise XOR operation, $x_{1}\left(0\right),y_{1}\left(0\right),x_{2}\left(0\right)$, and $y_{2}\left(0\right)$ are two groups of initial values of 2D-LASM chaotic system. $x_{1}^{{}^{\prime }}\left(0\right),y_{1}^{{}^{\prime }}\left(0\right),x_{2}^{{}^{\prime }}\left(0\right)$, and $y_{2}^{{}^{\prime }}\left(0\right)$ are two groups of initial values updated after the disturbance from MD5. Clearly, the new initial values will change with different plaintext images. The confusion sequence is generated by

(6) $$\left\{\begin{array}{c} L=H\times W\times Ch\\ L_{d}=L/2\\ R=[x_{1}\left(1:L_{d}\right);y_{1}\left(1:L_{d}\right)] \end{array}\right.$$

where R is composed of two chaotic sequences obtained by the initial value solution of 2D-LASM. The length of *R* is the same as L,H and *W* are the pixel rows and columns of the plaintext image to be encrypted, and Ch is the number of channels of the plaintext image to be encrypted. A complete colour image is composed of three channels of red, green, and blue, while a greyscale image requires only one channel. The mask obfuscation sequence is generated by

(7) $$\left\{\begin{array}{c} R_{c}^{{}^{\prime }}=flood\left(mod\left(R\times 10^{10},256\right)\right)\\ R_{c}=reshape\left(R_{c}^{{}^{\prime }},H,W\right) \end{array}\right.$$

where floor(∗) is a downward rounding operation; mod(∗) is a remainder function whose result is the remainder obtained by dividing two numerical expressions; reshape(∗) is a reshaping function generated Rc for the mask obfuscation operations, which is a sequence of integers with value range ∈ [0, 255], and its length is H×W. The pixel scrambling sequence is generated by

(8) $$\left\{\begin{array}{c} seq\_H=x_{2}\left(1:H\right)\\ seq\_W=y_{2}\left(2,1:8\times W\right)\\ {}[S_{1},R_{pr}]=sort\left(seq\_H\right)\\ {}[S_{2},R_{pc}]=sort\left(seq\_W\right) \end{array}\right.$$

where sort(∗) is the equation that sorts all elements of the sequence; seq_H denotes the chaos-based length sequence extracted from $x_{1}$; seq_W denotes the chaotic sequence of length 8 × W, extracted from $y_{2}$; $R_{pr}$ denotes a pixel row generated by the sorting function and length *H*; $R_{pc}$ denotes a pixel column generated by the sorting function with length 8 × W; and $S_{1}$ and $S_{2}$ are chaos-based sorted sequence values. The diffusion sequence is generated by

(9) $$\left\{\begin{array}{c} R_{d1}=flood\left(mod\left(R\times 10^{8},256\right)\right)\\ R_{d2}=flood\left(mod\left(R\times 10^{9},256\right)\right) \end{array}\right.$$

where the lengths of $R_{d1}$ and $R_{d2}$ are H×W. The $R_{d1}$ and $R_{d2}$ sequences are used for dynamic diffusion operations.

**Step 3**: Confusion

The ordinary image *P* is blurred with the confusion sequence $R_{c}$ so that the image can obtain the blurred image $C_{1}$ by hiding—namely,

(10) $$C_{1}\left(i\right)=R_{c}\left(i\right)⊕P\left(i\right),i=\left(1,2,\ldots ,L\right)$$

**Step 4**: Pixel permutation

The blurred image $C_{1}$ is encrypted by pixel dislocation using the dislocation sequence $R_{pc}\left(i\right)$ and $R_{pr}\left(j\right)$ to obtain image $C_{3}$—namely,

(11) $$\left\{\begin{array}{c} C_{2}=swap\left(C_{1}\left(:,R_{pc}\left(i\right)\right),C_{1}\left(:,i\right)\right)\\ C_{3}=swap\left(C_{2}\left(:,R_{pr}\left(j\right)\right),C_{1}\left(j,:\right)\right) \end{array}\right.$$

where i=1,2,…,H;j=1,2,…,8×W; $C_{2}$ is the image after the double-bit column transformation arrangement; $C_{3}$ is the image after the double-bit row transformation arrangement; and swap(∗) is used to exchange the values of two pixels.

**Step 5**: Dynamic diffusion

Dynamic diffusion allows each pixel to interact with each other, thus achieving an avalanche effect. By establishing a diffusion path between pixels and adding keys $R_{d1}$ and $R_{d2}$ to the diffusion process, the ciphertext pixels are diffused along that path to other pixels to generate the final ciphertext image *C*.

The diffusion encryption generation equation for the first greyscale pixel C(1) of the ciphertext image *C* is shown as

(12) $$\left\{\begin{array}{c} sum\left(1\right)=\sum_{i=1}^{L}C_{3}\left(i\right)\\ C\left(1\right)=C_{3}\left(1\right)⊕R_{d1}\left(1\right)⊕\left(sum\left(1\right)∔R_{d2}\left(1\right)\right) \end{array}\right.$$

where the operator ∔ can be defined as c∔d≜mod(c+d,256); $C_{3}\left(1\right)$ is the first pixel in the replacement image $C_{3}$; $R_{d1}\left(1\right)$ and $R_{d2}\left(1\right)$ are the first elements of the diffusion encryption sequence; and sum(1) represents the accumulation of all pixels of the replacement image $C_{3}$ and then generates the ciphertext pixel C(i)—namely,

(13) $$\left\{\begin{array}{c} C\left(i\right)=C_{3}\left(i\right)⊕\left(C\left(i-1\right)∔R_{d1}\left(i\right)⊕\left(sum\left(i\right)∔R_{d2}\left(i\right)\right)\right)\\ sum\left(i\right)=sum\left(i-1\right)-C_{3}\left(i\right) \end{array}\right.$$

where i=2,3,…,L; *i* denotes the *i*th pixel of image $C_{3}$ after remodelling; C(i−1) is the (i−1)th ciphertext pixel of the sequence of pixels representing dynamic diffusion encryption greyscale; and sum(i) is the cumulative sum of (L−i) pixel values of image $C_{3}$. According to Equation (13), starting from the second ciphertext pixel C(2), the cipher image *C* is generated by computing C(i) through iterations of *i* in {1,2,…,L}, until the *L*th ciphertext pixel C(L) is generated.

## 4. Experimental Verification and Discussion

### 4.1. Performance Analysis of the Image-Encryption Algorithm

The image-encryption algorithm proposed in this paper is based on the MATLAB r2018b system to complete the verification analysis. The system ran on a Windows 10 64-bit operating system, Intel(R) Core(TM) I7-6500U CPU @ 2.50 GHz 2.59 GHz processor and 8 GB memory running on a PC. This article selected some standard images as the test images for experiments, and most of the test images were from the “USC-SIPI Image Database [40]”.

#### 4.1.1. Key Space Analysis

The key space refers to the set of all possible keys that can be used to generate the key. The size of the key space depends on the length of the security key, which is one of the most important characteristics in determining the strength of a cryptosystem. The image-encryption algorithm designed in this paper uses a two-dimensional discrete chaotic system, and the key parameters involved are four chaotic initial values $x_{1}\left(0\right),y_{1}\left(0\right),x_{2}\left(0\right),$ and $y_{2}\left(0\right)$. The calculation accuracy of 64-bit double precision is $2^{15}$. The size of the key space of this part is $10^{15}\times 10^{15}\times 10^{15}\times 10^{15}=10^{60}\approx 2^{199}$.

Considering that the introduced MD5 can output 128-bit hash value, the key space of this encryption scheme is calculated as $2^{327}$, and the key length reaches 327 bits. From Table 1, it can be seen that the key space of this paper has obvious advantages compared with other existing encryption schemes, the key space of this paper has clear advantages. Therefore, the encryption algorithm in this paper can resist any form of brute force attack [41].

#### 4.1.2. Histogram Analysis

**Figure 7:**
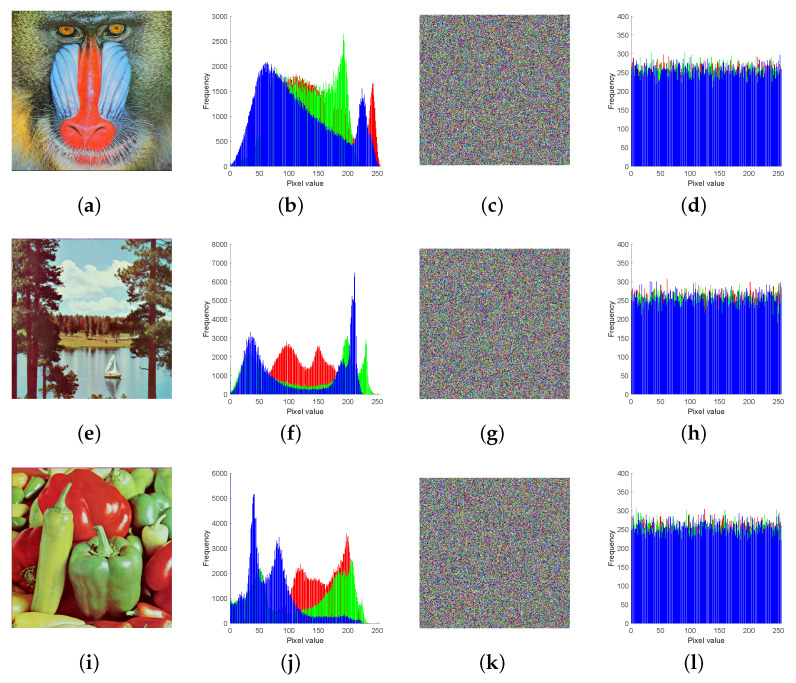
Histogram of images before and after encryption: (**a**) Plaintext image of “4.2.03.tiff”; (**b**) Histogram of the plaintext image of (**a**). (**c**) Ciphertext image of (**a**). (**d**) Histogram of the ciphertext image of (**a**). (**e**) Plaintext image of “4.2.06.tiff”. (**f**) Histogram of the plaintext image of (**e**); (**g**) Ciphertext image of (**e**). (**h**) Histogram of the ciphertext image of (**e**). (**i**) Plaintext image of “4.2.07.tiff”. (**j**) Histogram of the plaintext image of (**i**). (**k**) Ciphertext image of (**i**). (**l**) Histogram of the ciphertext image of (**i**).

The colour histogram is an important feature of the statistical properties of an image, with the horizontal coordinate indicating the pixel value and the vertical coordinate indicating how often the pixel appears in the image. The ideal encryption algorithm should result in different plaintext images having a uniform statistical distribution or a similar histogram that is independent of the plaintext image [44]. From Figure 7, we know that, although the histograms of the plaintext images are completely different, the histograms of the ciphertext images all have similar distribution characteristics, indicating that the algorithm has a strong resistance to statistical attacks.

#### 4.1.3. Coefficient of Adjacent Pixels

**Figure 8:**
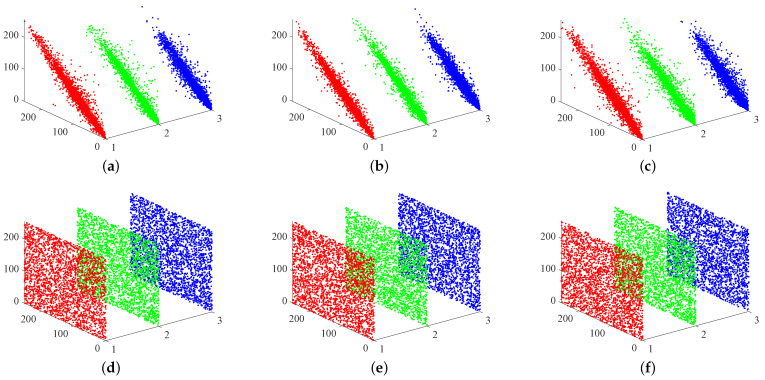
Correlation coefficients distribution map of plain image and ciphered image of “4.1.01.tiff”: (**a**) Correlation of “4.1.01.tiff” in the horizontal direction. (**b**) Correlation of “4.1.01.tiff” in the vertical direction. (**c**) Correlation of “4.1.01.tiff” in the diagonal direction. (**d**) Correlation of ciphered “4.1.01.tiff” in the horizontal direction. (**e**) Correlation of ciphered “4.1.01.tiff” in the vertical direction. (**f**) Correlation of ciphered “4.1.01.tiff” in the diagonal direction.

Every image has intrinsic and inherent characteristics, such as high pixel correlation and high redundancy [45]. Correlation analysis tests the strength of the correlation between image pixels, and the correlation between neighbouring pixels of a normal image is usually high, with a correlation coefficient closer to 1. At the same time, a secure and efficient encryption algorithm must satisfy the requirement of a low correlation coefficient between neighbouring pixels of a ciphertext image in order to resist statistical attacks. The correlation coefficient can be calculated from Equation (14)—namely,

(14) $$\left\{\begin{array}{c} E\left(x\right)=\frac{1}{N}\sum_{i=1}^{N}x_{i}\\ D\left(x\right)=\frac{1}{N}\sum_{i=1}^{N}{\left(x_{i}-E\left(x\right)\right)}^{2}\\ cov\left(x,y\right)=\frac{1}{N}\sum_{i=1}^{N}\left(x_{i}-E\left(x\right)\right)\left(y_{i}-E\left(y\right)\right)\\ \gamma_{xy}=\frac{cov(x,y)}{\sqrt{D(x)}\times \sqrt{D(y)}} \end{array}\right.$$

where the grey scale value of each pixel is denoted by *x* and *y*, respectively, E(x) denotes the mean value, D(x) denotes the variance, cov(x,y) denotes the covariance, and $\gamma_{xy}$ denotes the correlation coefficient. Table 2 shows the encryption quality of the proposed scheme and the classic encryption schemes in recent years. We evaluate the correlation between adjacent pixels in horizontal, vertical, and diagonal directions, as shown in Figure 8, from which it can be inferred that the correlation between adjacent pixels of the password is not strong. Therefore, from the experimental results, it can be found that the encryption algorithm in this paper can effectively resist statistical analysis [46].

#### 4.1.4. Analysis of Differential Attacks

**Figure 9:**
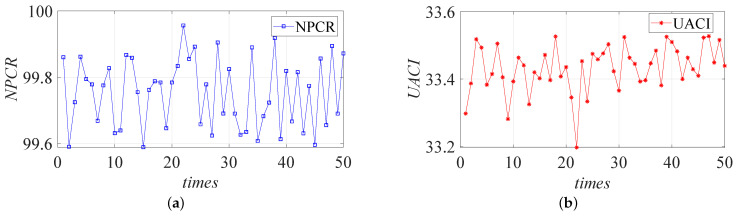
NPCR (**a**,**b**) values obtained from 50 experiments.

A complete encryption system should have a high sensitivity to images. The plaintext sensitivity can be one of the main indicators of the security of an encryption system and the strength of a cryptographic management system against differential attacks. Both the Number of Pixels Change Rate (NPCR) and the Unified Average Changing Intensity (UACI) can be used to represent the difference between two images that change only on the same pixel [49]. The formula of NPCR and UACI is defined by

(15) $$\left\{\begin{array}{c} NPCR=\frac{1}{H\times W}\times \sum_{i=1}^{H}\sum_{j=1}^{W}D\left(i,j\right)\times 100\%\\ UACI=\frac{1}{H\times W}\times \sum_{i=1}^{H}\sum_{j=1}^{W}\frac{|v_{1}\left(i,j\right)-v_{2}\left(i,j\right)|}{255}\times 100\%\\ D\left(i,j\right)=\left\{\begin{array}{c} 0,v_{1}\left(i,j\right)=v_{2}\left(i,j\right)\\ 1,v_{1}\left(i,j\right)\neq v_{2}\left(i,j\right) \end{array}\right. \end{array}\right.$$

where $v_{1}\left(i,j\right)$ denotes the pixel value of a ciphertext pixel before it is changed; and $v_{2}\left(i,j\right)$ denotes the pixel value of a ciphertext image after changing the pixel value at a point in the plaintext image. We compare the values of NPCR and UACI in this paper with other works in Table 3. In addition, the values of NPCR and UACI after 50 experiments are shown in Figure 9. The NPCR and UACI average values are near the theoretical value; thus, the encryption algorithm in this paper is sensitive to the pixel changes in the plaintext image. Therefore, the encryption algorithm designed in this paper can effectively resist differential attacks.

#### 4.1.5. Image Quality Analysis

The Peak Signal-to-Noise Ratio (PSNR) and Structural SIMilarity (SSIM) are commonly used in the field of image processing as a tool to weigh the quality of encryption. The Mean Square Error (MSE) is a part of PSNR and is defined by

(16) $$\left\{\begin{array}{c} MSE=\frac{1}{H\times W}\sum_{i=1}^{H}\sum_{j=1}^{W}{\left(X(i,j)-Y(i,j)\right)}^{2}\\ PSNR=10\times \log_{10}\left(\frac{Q^{2}}{MSE}\right) \end{array}\right.$$

where MSE denotes the mean square error of the plaintext image *X* and the ciphertext image *Y*. The height and width of the image are denoted by *H* and *W*, respectively, and *Q* denotes the pixel level of the image. SSIM is a measure of the similarity of two images and is defined by

(17) $$SSIM\left(X,Y\right)=\frac{\left(2\mu {}_{X}\mu {}_{Y}+{\left(0.01L\right)}^{2}\right)\left(2\sigma_{XY}+{\left(0.03L\right)}^{2}\right)}{\left(u_{X}^{2}+u_{Y}^{2}+{\left(0.01L\right)}^{2}\right)\left(\sigma {}_{X}^{2}+\sigma {}_{Y}^{2}+{\left(0.03L\right)}^{2}\right)}$$

where $\mu {}_{X}$, $\mu_{Y}$ denote the mean of image *X* and *Y*, respectively, $\sigma_{X}$, $\sigma_{Y}$ denote the standard deviation of image *X* and *Y*, respectively, and *L* denotes the dynamic range of pixel values. The values of PSNR and SSIM are calculated by using Equations (16) and (17) as shown in Table 4. The experimental results show that the PSNR and SSIM values obtained by this algorithm are low. Therefore, this encryption scheme has certain advantages.

#### 4.1.6. Sensitivity Analysis

**Figure 10:**
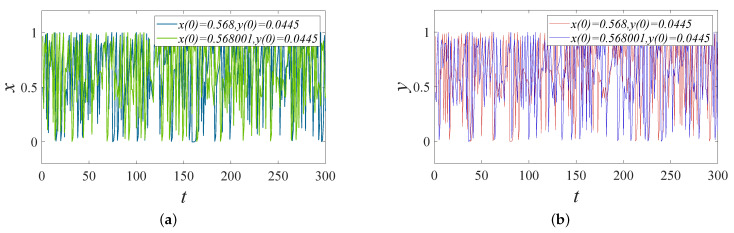
Key sequence sensitivity timing diagram: (**a**) Comparison x before and after x(0) key perturbation. (**b**) Comparison y before and after x(0) key perturbation.

In image encryption, key sensitivity performance is often used as an important indicator to measure the security of an encryption system. The key sensitivity is generally expressed by the difference between corresponding images when decrypting or encrypting the same image with a slightly different key. In order to test the sensitivity to the key in the scheme, we processed the two chaotic series by means of time series and compared the generated two-dimensional chaotic series. It can be seen from Figure 10 that the encryption system designed in this paper has high security and strong sensitivity to keys, which increases the difficulties for attackers to decipher the ciphered image.

#### 4.1.7. Information Entropy

Information entropy is an indicator of the amount and uncertainty of information contained in digital images. The information entropy of the image is positively correlated with the encryption effect. The larger the information entropy is, the better effect the encryption will have. The formula of information entropy is defined by

(18) $$H\left(n\right)=-\sum_{i=1}^{L}P\left(n_{i}\right)log_{2}P\left(n_{i}\right)$$

where *i* represents the pixel greyscale value, and P(i) represents the probability that this grey value will appear in the digital image. From the calculation of Equation (18), the theoretical value of information entropy is the maximum value of 8. We compare the information entropy of the images before and after encryption, and the experimental result is shown in Table 5. It can be seen that the information entropy of the two ciphertext images is above 7.9994, which has a certain improvement compared with the similar references.

#### 4.1.8. Efficiency Analysis

There are many factors that can affect the efficiency, such as the size of the image, the degree of arithmetic power consumed by the encryption operation. We selected the images with sizes of 256 × 256, 512 × 512, and 1024 × 1024 for comparison [50]. As shown in Table 6, when the image size becomes larger, the required encryption and decryption time will increase accordingly. From the experimental results, it can be seen that the algorithm has high encryption efficiency.

### 4.2. Embedded Hardware Implementation of Image Encryption in Optical Access Network

**Figure 11:**
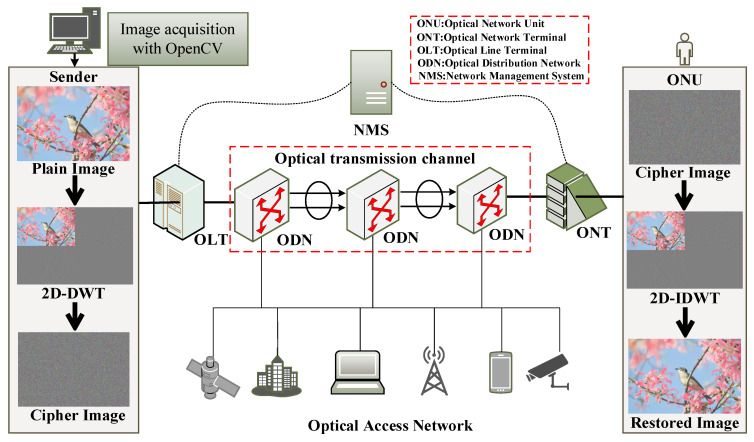
Embedded implementation of image wavelet transform encryption used in an Automatic Switched Optical Network.

The image encryption system can also be effectively applied in the optical access network communication scenario, which is illustrated in Figure 11. In this scenario, both the sender and the receiver are embedded terminals for reading and displaying, sending and receiving, and encrypting and decrypting images. In order to enhance the security of the information, we use the optical fibre transmission method to replace the traditional ordinary information transmission method, which greatly enhances the information transmission process while ensuring real-time performance. The possibility of protection from dangerous elements during information transmission is greatly enhanced. Therefore, the encryption method that we propose is suitable for secure communication in an optical access network environment.

To verify the effectiveness and feasibility of the image encryption system, we conducted experimental analysis on an experimental platform of optical access network based on ARM embedded system. The security, encryption speed, resource utilisation, and cracking difficulty of hardware encryption are all greatly superior to software encryption. The optical access network-based digital image encryption communication system consists of two ARM chip-based embedded development boards and a Gigabit single-mode single-fibre transceiver, TP-LINKTL-FC311A-3.

The maximum transmission distance is approximately 10 km and the maximum transmission rate is above 155 Mbit/s. The ARM development board is the Raspberry Pi 4B, and the programming language used is Python. The chip is a Broadcom BCM2711 with Cortex-A72 architecture, and the operating system is 32-bit Linux 5.4 with a 3.5-inch liquid crystal display (LCD). The wireless router is used for network communication between the sender and the receiver and obtains the sending and receiving addresses via Dynamic Host Configuration Protocol (DHCP), 192.168.1.114 and 192.168.1.115, respectively.

**Figure 12:**
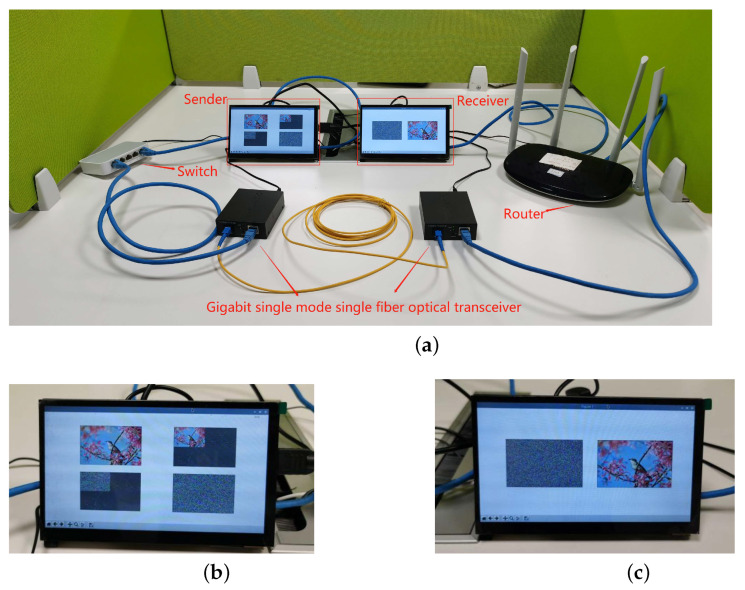
Experimental results in an Automatic Switched Optical Network secure communication platform: (**a**) The overall physical diagram. (**b**) Image encryption. (**c**) Decryption image of (**b**).

**Figure 13:**
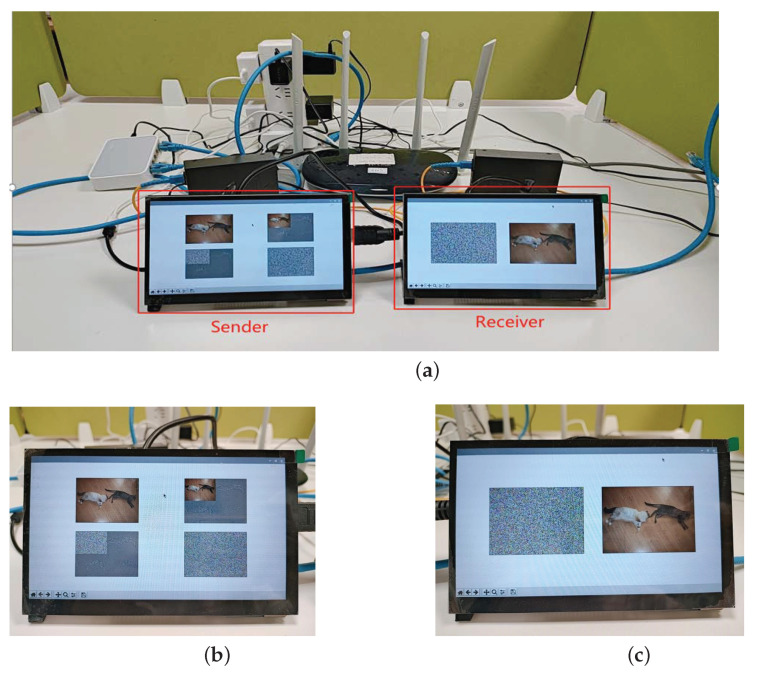
Experimental results in an Automatic Switched Optical Network secure communication platform: (**a**) The overall physical diagram. (**b**) Image encryption. (**c**) Decryption image of (**b**).

The sender is responsible for reading, displaying, encrypting, and transmitting the plaintext images, while the receiver is responsible for receiving, displaying, and decrypting the corresponding ciphertext images. The experimental platform and results are shown in Figure 12 and Figure 13.

## 5. Conclusions

This paper proposed an image encryption scheme based on 2D-LASM and DWT and completed corresponding embedded hardware experiments. To enhance the security performance, we designed an MD5 plaintext association mechanism for obtaining dynamic chaotic sequences to resist the chosen plaintext attacks. In terms of specific encryption operations, we first performed Discrete Wavelet Transform (DWT) on the plaintext image to decompose low-frequency (LF) coefficients and high-frequency (HF) coefficients and encrypted them in different ways according to the weights.

Then, we used the chaotic sequences to perform dynamic diffusion operations on the reconstructed intermediate ciphertext frequency-domain image to obtain the final cipher image. Furthermore, the proposed encryption algorithm was successfully tested in an embedded optical access network communication environment. Theoretical analysis and simulation experiments showed that the algorithm has the characteristics of large key space, excellent statistical analysis performance and the ability to resist various common attacks.

Although the frequency processing is relatively complicated, the encryption algorithm still has the advantages of easy implementation and high efficiency. At the same time, its successful implementation on embedded devices in the optical network environment demonstrated that the algorithm has certain practicability in an Internet-of-Things environment. 

## Figures and Tables

**Table 1:** Key space size comparison table.

	This Article	Ref. [42]	Ref. [43]
Key space/bits	327	128	309

**Table 2:** Comparison results of the correlation coefficients of adjacent pixels.

Component	Direction	Original Images	Algorithm in This Paper	Ref. [47]	Ref. [48]	Ref. [9]
R	Horizontal	0.9736	−0.0215	−0.0063	0.0076	0.0023
	Vertical	0.934	−0.0219	−0.0016	0.0017	−0.0130
	Diagonal	0.934	0.0082	0.00156	0.0110	−0.0061
G	Horizontal	0.96	0.0272	−0.0032	−0.0048	−0.0236
	Vertical	0.9346	0.0263	0.0335	0.0274	0.0308
	Diagonal	0.9419	0.005	−0.0095	0.0342	−0.0179
B	Horizontal	0.9624	0.0286	−0.0044	−0.0056	−0.0266
	Vertical	0.9213	−0.0489	−0.0079	0.0150	−0.0057
	Diagonal	0.8979	0.0155	0.0034	−0.0115	0.0378

**Table 3:** Differential attack analysis.

Images	NPCR(%)				UACI(%)			
	This Paper	Ref. [47]	Ref. [48]	Ref. [9]	This Paper	Ref. [47]	Ref. [48]	Ref. [9]
4.2.06.tiff [40]	99.6758	99.6188	99.6269	99.6033	33.4371	24.8663	27.9449	32.2283
4.2.03.tiff [40]	99.8474	99.6081	99.6189	99.6057	33.5871	29.9629	29.9636	29.9411
7.1.02.tiff [40]	99.8426	99.6020	99.5995	99.6132	33.4306	29.3349	29.3636	29.3176
2.2.03.tiff [40]	99.6282	99.6172	99.6072	99.6144	33.4007	31.2027	27.6087	31.2021

**Table 4:** Image quality analysis.

Images	Original Image and Ciphered Image			Original Image and Restored Image	
	PSNR(dB)	MSE	SSIM	PSNR(dB)	MSE
4.1.07.tiff [40]	25.6436	177.3049	0.0106	39.6267	7.0862
5.1.11.tiff [40]	25.5066	182.9874	0.011	39.593	7.1413
5.1.13.tiff [40]	24.6653	222.0984	0.0064	38.3199	11.418

**Table 5:** Image information entropy.

Images	R	G	B
Original image	7.5549	7.0167	6.7347
Ciphertext image in this paper	7.9994	7.9994	7.9993
Ref. [47]	7.9993	7.9994	7.9994
Ref. [48]	7.9992	7.9993	7.9992
Ref. [9]	7.9993	7.9993	7.9993

**Table 6:** Encryption time comparison.

Images	Encryption Time (s)	Decryption Time (s)
4.1.01.tiff (256 × 256)	0.599185	0.078700
4.2.03.tiff (512 × 512)	0.417543	0.199085
2.2.02.tiff (1024 × 1024)	1.239165	0.707843
2.2.03.tiff (1024 × 1024)	1.168449	0.646701

## Data Availability

Data sharing not applicable to this article as no datasets were generated during the current study.
